# Impact of trauma system structure on injury outcomes: a systematic review protocol

**DOI:** 10.1186/s13643-017-0408-8

**Published:** 2017-01-21

**Authors:** Lynne Moore, Howard Champion, Gerard O’Reilly, Ari Leppaniemi, Peter Cameron, Cameron Palmer, Fikri M. Abu-Zidan, Belinda Gabbe, Christine Gaarder, Natalie Yanchar, Henry Thomas Stelfox, Raul Coimbra, John Kortbeek, Vanessa Noonan, Amy Gunning, Luke Leenan, Malcolm Gordon, Monty Khajanchi, Michèle Shemilt, Valérie Porgo, Alexis F. Turgeon, on behalf of the International Injury Care Improvement Initiative

**Affiliations:** 1 Department of Social and Preventative Medicine, Université Laval, Québec, QC Canada; 2 Axe Santé des Populations et Pratiques Optimales en Santé (Population Health and Optimal Health Practices Research Unit), Traumatologie–Urgence-Soins intensifs (Trauma–Emergency–Critical Care Medicine), CHU de Québec–Université Laval Research Center (Enfant-Jésus Hospital), 1401, 18e rue, local H-012a, Québec, G1J 1Z4 Canada; 3 U Health Sciences, Baltimore, Maryland USA; 4 Emergency and Trauma Centre, The Alfred Hospital, Melbourne, Australia; 5 School of Public Health and Preventive Medicine, Monash University, Melbourne, Australia; 6 Department of Surgery, Helsinki University, Helsinki, Finland; 7 Emergency and Trauma Centre, The Alfred Hospital, Monash University, Melbourne, Australia; 8 Trauma Service, Royal Children’s Hospital, Melbourne, Australia; 9 Department of Surgery, College of Medicine and Health Sciences, UAE University, Al-Ain, United Arab Emirates; 10 Department of Traumatology, Oslo University Hospital Ulleval, Oslo, Norway; 11 Department of Surgery, Dalhousie University, Halifax, Nova Scotia Canada; 12 Departments of Critical Care Medicine, Medicine and Community Health Sciences, O’Brien Institute for Public Health, University of Calgary, Calgary, Canada; 13 Division of Trauma, Surgical Critical Care, Burns, and Acute Care Surgery, University of California, San Diego Health System, San Diego, California USA; 14 Department of Surgery, Division of General Surgery and Division of Critical Care, University of Calgary, Calgary, Alberta Canada; 15 Rick Hansen Institute, Vancouver, BC Canada; 16 Department of Surgery, University Medical Center Utrecht, Utrecht, The Netherlands; 17 Department of Emergency Medicine, University of Glasgow, Glasgow, UK; 18 Seth G.S. Medical College and KEM Hospital, Mumbai, India

**Keywords:** Trauma system, Organizational-level intervention, Injury outcomes

## Abstract

**Background:**

Injury represents one of the greatest public health challenges of our time with over 5 million deaths and 100 million people temporarily or permanently disabled every year worldwide. The effectiveness of trauma systems in decreasing injury mortality and morbidity has been well demonstrated. However, the organisation of trauma care varies significantly across trauma systems and we know little about which components of trauma systems contribute to their effectiveness. The objective of the study described in this protocol is to systematically review evidence of the impact of trauma system components on clinically significant outcomes including mortality, function and disability, quality of life, and resource utilization.

**Methods:**

We will perform a systematic review of studies evaluating the association between at least one trauma system component (e.g. accreditation by a central agency, interfacility transfer agreements) and at least one injury outcome (e.g. mortality, disability, resource use). We will search MEDLINE, EMBASE, COCHRANE central, and BIOSIS/Web of Knowledge databases, thesis holdings, key injury organisation websites and conference proceedings for eligible studies. Pairs of independent reviewers will evaluate studies for eligibility and extract data from included articles. Methodological quality will be evaluated using elements of the ROBINS-I tool and the Cochrane risk of bias tool for non-randomized and randomized studies, respectively. Strength of evidence will be evaluated using the GRADE tool.

**Discussion:**

We expect to advance knowledge on the components of trauma systems that contribute to their effectiveness. This may lead to recommendations on trauma system structure that will help policy-makers make informed decisions as to where resources should be focused. The review may also lead to specific recommendations for future research efforts.

**Systematic review registration:**

This protocol was registered with the International Prospective Register of Systematic Reviews (PROSPERO) on 28-06-2016. PROSPERO 2016:CRD42016041336 Available from http://www.crd.york.ac.uk/PROSPERO/display_record.asp?ID=CRD42016041336.

**Electronic supplementary material:**

The online version of this article (doi:10.1186/s13643-017-0408-8) contains supplementary material, which is available to authorized users.

## Background

Injury is the leading cause of death under 40 years of age, the leading cause of loss of active life years and is second only to cardiovascular diseases in terms of healthcare costs in high-income countries [1, 2]. Low- to middle-income countries carry more than 90% of the burden of injury deaths [3, 4]. Important reductions in injury mortality, disability, and costs have been achieved in many healthcare jurisdictions with the introduction of trauma systems [5–9].

There are many definitions of a trauma system (see Table 1 for examples). Broadly, a trauma system is an organized, regional, multidisciplinary response to injury. Most definitions focus on tertiary prevention in the public health model, i.e. reducing the consequences of an injury that has already occurred. It is important to note that the complete public health model also embodies primary prevention (preventing injury before it occurs, e.g. speeding legislation) and secondary prevention (mitigation of the effects at the time of injury, e.g. functional air bags) [10]. While all of these elements are critical to effective injury control, [11 12] this review will focus on tertiary injury prevention.

**Table 1:** Examples of trauma system definitions from injury control organizations around the world

Organisation	Definition
World Health Organization http://www.who.int/emergencycare/gaci/gaci_flyer_web.pdf?ua=1%5d	A preplanned approach to the provision of the spectrum of trauma services, including but not limited to injury prevention and control initiatives, timely transport from scene of injury to trauma care facility, availability of trauma care providers and services when needed, and rehabilitation
US Department of Health and Human Services http://www.emsa.ca.gov/Media/Default/Word/ModelTraumaSystemPlanningAndEvaluation.pdf	Preplanned, comprehensive, and coordinated statewide and local injury response networks that include all facilities with the capability of care for the injured.
US National Highway Traffic Safety Administration http://www.nhtsa.gov/people/injury/ems/emstraumasystem03/traumasystem.htm	Organized, coordinated effort in a defined geographic area that delivers the full range of care to all injured patients and is integrated with the local public health system
Australian Institute of Health and Welfare http://www.aihw.gov.au/burden-of-disease/	Integrated and systematic structure designed to facilitate and coordinate a multidisciplinary system response to provide optimal care to injured patients from onset of injury through rehabilitation and return of ideal functioning
Trauma Association of Canada http://www.traumacanada.ca/accreditation_committee/Accreditation_Guidelines_2011.pdf	A preplanned, organized, and coordinated injury control effort in a defined geographic area
UK Trauma network http://www.nhs.uk/NHSEngland/AboutNHSservices/Emergencyandurgentcareservices/Pages/Majortraumaservices.aspx	A model of care designed to care for patients with multiple serious injuries that could result in death or serious disability, including head injuries, life-threatening wounds and multiple fractures
European commission http://ec.europa.eu/transport/road_safety/specialist/knowledge/postimpact/trauma_care/establishing_a_national_trauma_system_en.htm	In a model system, key trauma system elements (Leadership, Professional resources, Education and advocacy, Information, Finances, Research, Technology, Disaster preparedness and response) are integrated and coordinated to provide cost-efficient and appropriate services
State of Israel trauma model http://www.ncbi.nlm.nih.gov/pmc/articles/PMC3634231/	A chain of arrangements and preparedness to provide quality response to injured from the site of injury to the appropriate hospital for the full range of care

Many injury organisations, including the World Health Organization (WHO) [11] and the American College of Surgeons (ACS), [12] emit consensus-based recommendations on the structure of trauma systems (see Table 2). Consequently, system components such as pre-hospital triage and transport protocols, accreditation and designation, and benchmarking activities as well as their level of integration vary significantly across trauma systems [12]. The effectiveness of trauma systems has now been well established, i.e. they have been associated with improvements in clinically important outcomes [13]. However, there is still a major knowledge gap on which components of a trauma system contribute to their effectiveness.

**Table 2:** Recommended trauma system components based on World Health Organisation [11] and American College of Surgeons criteria [13]

Core components	Subcomponents
Oversight	Trauma system plan Trauma system advisory committee Trauma services medical director Lead agency Disaster planning
Prehospital care	Pre-hospital major trauma definition EMS transport system Triage and transport protocols Emergency services medical director EMS treatment protocols Communication between EMS and hospitals
Definitive care	Facility designation through an accreditation agency Inclusive design Sitting of facilities (coverage) Interfacility transfer agreements/protocols Communication between transferring hospitals
Rehabilitation	Integrated rehabilitation services
Human resources	Workforce resources Educational preparation
Evaluation	Data collection–trauma registries Injury surveillance Benchmarking Integration of evaluation throughout the care continuum Research Interdisciplinary review committee

Our aim is to systematically review evidence of the impact of trauma system components on clinically significant injury outcomes including mortality, function and disability, quality of life, and resource utilization. Our systematic review protocol was registered with the International Prospective Register of Systematic Reviews (PROSPERO) on 20-06-2016 (#42016041336). The review will be conducted between 01-11-2016 and 21-02-2017.

## Methods

We will conduct a systematic review in accordance with Cochrane guidelines [14]. The protocol is presented using the structure suggested in Preferred reporting items for systematic review and meta-analysis protocols (PRISMA-P) 2015 (Additional file 1) [15]. Any important protocol amendments will be recorded in PROSPERO and reported in the systematic review manuscript.

### Eligibility criteria

#### Study designs

We will consider randomized and non-randomized controlled trials (RCT) including cluster RCT, interrupted time series studies, controlled before-after studies, and prospective or retrospective observational studies. We will also include studies based on qualitative methods (e.g. preventable death determined by expert consensus).

#### Participants

We will include studies based on injury populations at large as well as studies evaluating population-based injury outcomes (e.g. population rates of injury mortality or hospitalization). No restrictions will be placed on age, injury type, or injury severity. Studies based exclusively on combat injuries, isolated fractures following low falls, burns, bites, foreign bodies, or late effects of injuries will be excluded.

#### Interventions

We will include studies evaluating the effectiveness of trauma system components, i.e. organizational-level structural interventions (single or multiple) related to tertiary injury prevention (see Table 2 for examples based on WHO and ACS guidelines). Interventions will be classified as using an adaptation of WHO and ACS categories, i.e. oversight, pre-hospital care, definitive care, rehabilitation, and evaluation.

#### Comparators

We will include studies comparing a single or multiple organizational-level structural intervention to either (i) usual trauma system structure or (ii) an alternative organizational structure. In order to be as inclusive as possible and given the variation in definitions of trauma systems (Table 1), we will include studies based on authors’ definition of a trauma system and document that definition. No restrictions will be based on the country or the regulatory nature of the trauma system (e.g. mandatory, non-mandatory, or volunteer). Studies comparing health care jurisdictions with a trauma system to those without organized trauma care will not be included as evidence of the global effectiveness of trauma systems has already been reviewed [13].

#### Outcome measures

Endpoints of interest will be clinically significant outcomes related to mortality, function and disability, quality of life, and resource utilization including the following:

- Primary endpoints:
  - Mortality (e.g. population-based, in-hospital, 30 days, 12 months, preventable)
  - Functional capacity (e.g. Functional Independence Measure, Glasgow Outcome Scale, return to work, level of dependency)
  - Quality of life (e.g. Short Form-36, WHOQOL, EQ-5D)
  - Burden of disease (e.g. years of life lost, Quality-Adjusted Life Years, Disability-Adjusted Life Years)
- Secondary endpoints:
  - Adverse events (complications, patient safety events)
  - Healthcare utilization (e.g. hospital, intensive care unit, ventilator, and rehabilitation length of stay; hospital readmission; emergency department visits; general practitioner visits; paramedical services)
  - Costs (e.g. acute care, rehabilitation, loss in productivity)

No restrictions will be set on the follow-up of patients for the evaluation of injury outcomes. No language restrictions will be used.

### Information sources

The search strategy is designed to minimize publication bias, including geographical bias. We will systematically search MEDLINE, EMBASE, COCHRANE central, and BIOSIS/Web of Knowledge databases from their inception up to a maximum of 9 months before publication submission for eligible studies. Unpublished clinical studies will be searched using ClinicalTrials and the ISRCTN registry. We will consult thesis repositories to identify additional studies, including Thesis portal Canada, EtHOS, DART-Europe E-Theses Portal, the National Library of Australia’s TROVE and ProQuest Dissertations & Theses Global. We will also search the websites of key healthcare organizations (WHO, public health agencies) and injury organisations including the American College of Surgeons, the Trauma Association of Canada, the International Association for Trauma Surgery and Intensive Care, the Australasian Trauma Society and the Trauma Audit Research Network. We will then screen references of included articles and abstracts of major injury conferences including the International Surgical Week, World Congress of Surgery, American Association for the Surgery of Trauma Congress, European Congress of Trauma and Emergency Surgery, World Trauma Congress, Eastern Society for the Surgery of Trauma Congress, Trauma Association of Canada annual meeting, and Australasian Trauma Society Congress.

### Search strategy

We will develop a rigorous systematic search strategy with a health sciences librarian who has systematic review experience using published guidelines of The Cochrane Collaboration (see Additional file 2 for a preliminary version) [16]. The strategy will be developed for MEDLINE and EMBASE using keywords and MeSH (MEDLINE) or EMTREE (EMBASE). To be as inclusive as possible, we will limit the search strategy to terms covering the concept of trauma system. Keywords will be elaborated by co-investigators and collaborators with methodological and clinical expertise. This search strategy will then be adapted to the other databases. The health sciences librarian will conduct a peer review of the search strategy using the Peer Review of Electronic Search Strategies (PRESS) checklist [17].

### Study records

#### Data management

Citations will be managed using EndNote software (version X7.0.1, New York City: Thomson Reuters, 2011). Duplicates will be identified and eliminated using electronic and manual screening. Multiple publications based on the same data will be identified by crosschecking authors, dates, and settings. We will identify only one publication for analyses using criteria based on study dates (most recent), sample size (largest), correspondence with inclusion criteria (highest correspondence), and risk of bias (lowest risk).

#### Selection process

Pairs of reviewers will independently evaluate citations for potential inclusion by screening titles and abstracts and will assess full publications to determine eligibility for final inclusion. To ensure high agreement on study eligibility, three samples of 500 citations will be independently and consecutively assessed by each reviewer. Between each assessment, results will be discussed to reach a consensus on the interpretation of inclusion criteria. Any further disagreement on study eligibility will be resolved by consensus, and a third reviewer will adjudicate if necessary. If information on eligibility is unavailable or unclear, study authors will be contacted to clarify.

#### Data collection

A standard electronic data abstraction form and a detailed instruction manual will be developed and piloted on a representative sample of five studies (Additional file 3). Pairs of reviewers with methodological and content expertise will independently extract information on study setting and design, study population, interventions, outcomes, measures of association with standard errors, and risk adjustment. Abstracts from conference proceedings will be included if they provide information on all of the above.

### Risk of bias in individual studies

Risk of bias in observational studies will be evaluated using the ROBINS-I risk of bias assessment tool for non-randomized studies [18]. The tool evaluates baseline and time-varying confounding, co-interventions, selection bias, classification bias (intervention), missing data, and bias in outcome measurement. If any randomized controlled trials are identified, risk of bias will be evaluated using the Cochrane risk of bias tool [19]. Two reviewers will independently evaluate risk of bias and rate studies using respective tools (low, moderate serious, critical, unclear for the ROBINS-I tool and high, low, unclear for the Cochrane risk of bias tool). Disagreement will be resolved using arbitration by a third reviewer.

#### Measures of treatment effect

For dichotomous outcomes, data will be summarized using odds ratios (OR) or risk ratios (RR), depending on data available and their 95% confidence intervals (CI). For continuous outcomes, we will use weighted or standardized mean difference.

#### Assessment of heterogeneity

Statistical heterogeneity will be measured using I^2^ statistics [14]. Heterogeneity will be interpreted as low from 0 to 40%, moderate from 30 to 60%, substantial from 50 to 90% and considerable from 75 to 100% [14].

### Data synthesis

If three or more studies have evaluated the same intervention or category of intervention (oversight, prehospital care, definitive care, rehabilitation, evaluation) and the same outcome, we will calculate pooled effect estimates and their 95% confidence intervals using random effects models adapted to the scale of measurement. We will use Review Manager (RevMan) (version 5.1, Copenhagen: The Nordic Cochrane Centre, The Cochrane Collaboration, 2012). If heterogeneity across studies in terms of populations, design, methods, intervention, and/or outcome (s) is too great to perform meta-analyses, we will present results using a systematic narrative synthesis. We will explore the results according to categories of interventions and outcomes taking account of risk of bias, in line with *Centre for Reviews and Dissemination* recommendations [20]. The narrative will be written by the lead reviewer and then checked independently by at least one other reviewer. Any disagreements will be adjudicated by a third reviewer.

### Subgroup and sensitivity analyses

If the number of studies is sufficient (≥3), we will perform subgroup analyses by age group, injury severity, and injury type (e.g. traumatic brain injury, spinal cord injury), according to authors’ definitions. We will also stratify by length of follow-up, World Bank country economic classifications (low–middle income, high income) and conduct sensitivity analyses excluding studies of low methodological quality.

### Publication bias

We will visually inspect funnel plots to evaluate the risk of publication bias.

### Strength of the body of evidence

We will evaluate the quality of evidence and strength of recommendations using the *Grading of Recommendations Assessment, Development and Evaluation* (GRADE) working group methodology [21].

## Discussion

Organised trauma systems require significant human and financial resources. Understanding which components of trauma system structure favor optimal injury outcomes is critical to ensure policy-makers can make informed decisions as to where resources should be focused. This systematic review will be a first step to identifying which components of trauma systems relative to tertiary injury care drive optimal outcomes. If summarized evidence is strong enough, [21] our quantitative or qualitative synthesis may lead to tentative recommendations on trauma system structure and on specific areas for future research.

## Additional files

Additional file 1: PRISMA-P (Preferred Reporting Items for Systematic review and Meta-Analysis Protocols) 2015 checklist. Recommended items to address in a systematic review protocol. (PDF 104 kb)
Additional file 2: Search strategy for MEDLINE and EMBASE. Tables describing preliminary search strategy and results for MEDLINE and EMBASE searches. (PDF 102 kb)
Additional file 3: Data collection form. Form for extracting data from eligible articles. (PDF 145 kb)

## Abbreviations

ACS: American College of Surgeons
CI: Confidence intervals
GRADE: Grading of Recommendations Assessment, Development and Evaluation
OR: Odds ratios
PRESS: Peer Review of Electronic Search Strategies
PROSPERO: International Prospective Register of Systematic Reviews
RCT: Randomized controlled trials
RR: Risk ratios
WHO: World Health Organization

## References

[1] HCUP Facts and Figures: Statistics on hospital-based Care in the United States, 2007. Agency for Healthcare Research and Quality, 2009. Available at: https://www.hcup-us.ahrq.gov/reports/factsandfigures.jsp. Accessed 17 Jan 2017. 21595104

[2] The cost of injury in Canada. Parachute Canada 2015. Available at: http://www.parachutecanada.org/costofinjury. Accessed 17 Jan 2017.

[3] Hofman K, Primack A, Keusch G (2005). Addressing the growing burden of trauma and injury in low- and middle-income countries. Am J Public Health.

[4] Mock CN, Jurkovich GJ, Nii-Amon-Kotei D (1998). Trauma mortality patterns in three nations at different economic levels: implications for global trauma system development. J Trauma.

[5] Mackenzie EJ, Rivara FP, Jurkovich GJ (2007). The national study on costs and outcomes of trauma. J Trauma.

[6] Moore L, Turgeon A, Lauzier F (2015). Evolution of patient outcomes over 14 years in a mature, inclusive Canadian trauma system. World J Surg.

[7] Gabbe BJ, Biostat GD, Lecky FE (2011). The effect of an organized trauma system on mortality in major trauma involving serious head injury: a comparison of the United kingdom and Victoria, Australia. Ann Surg.

[8] Gabbe BJ, Lyons RA, Fitzgerald MC (2015). Reduced population burden of road transport-related major trauma after introduction of an inclusive trauma system. Ann Surg.

[9] Gabbe BJ, Simpson PM, Sutherland AM (2012). Improved functional outcomes for major trauma patients in a regionalized, inclusive trauma system. Ann Surg.

[10] Haddon W (1970). On the escape of tigers: an ecologic note. Am J Public Health Nations Health.

[11] Moore L, Evans D, Hameed SM, et al. Mortality in Canadian trauma systems: a multicenter Cohort Study. Ann Surg. 2016 Jan 7 [Epub ahead of print]. 10.1097/SLA.0000000000001614 28009748

[12] American College of Surgeons Committee on Trauma (2008). Regional trauma systems: optimal elements, integration, and assessment. Systems consultation guide.

[13] Celso B, Tepas J, Langland-Orban B (2006). A systematic review and meta-analysis comparing outcome of severely injured patients treated in trauma centers following the establishment of trauma systems. J Trauma.

[14] Kennedy MP, Gabbe BJ, McKenzie BA (2015). Impact of the introduction of an integrated adult retrieval service on major trauma outcomes. Emerg Med J.

[15] Shamseer L, Moher D, Clarke M (2015). Preferred reporting items for systematic review and meta-analysis protocols (PRISMA-P) 2015: elaboration and explanation. BMJ.

[16] Simon R, Stone M, Cucuzzo J (2009). The impact of a new trauma center on an existing nearby trauma center. J Trauma Inj Infect Crit Care.

[17] Sampson M, Mcgowan J, Cogo E (2009). An evidence-based practice guideline for the peer review of electronic search strategies. J Clin Epidemiol.

[18] Claridge JA, Allen D, Patterson B (2013). Regional collaboration across hospital systems to develop and implement trauma protocols saves lives within 2 years. Surgery (United States).

[19] Higgins JP, Altman DG, Gotzsche PC (2011). The Cochrane Collaboration’s tool for assessing risk of bias in randomised trials. BMJ.

[20] Eastman AB (2010). Wherever the dart lands: Toward the ideal trauma system. J Am Coll Surg.

[21] Grading the strength of a body of evidence when assessing health care interventions for the Effective Health Care Program of the Agency for Healthcare Research and Quality: An Update. Agency for Healthcare Research and Quality, 2008. Available at: http://effectivehealthcare.ahrq.gov/index.cfm/search-for-guides-reviews-and-reports/?pageaction=displayproduct&productid=1752. Accessed 17 Jan 2017. 24404627

