# Supplementary material for: Impact of trauma system structure on injury outcomes: a systematic review protocol
Source: Syst Rev. 2017 Jan 21;6:12. doi: 10.1186/s13643-017-0408-8 (PMC5251247; doi:10.1186/s13643-017-0408-8)
Supplement: Additional file 2: — Search strategy for MEDLINE and EMBASE. Tables describing preliminary search strategy and results for MEDLINE and EMBASE searches. (PDF 102 kb) [file 13643_2017_408_MOESM2_ESM.pdf]

Search strategy for MEDLINE and EMBASE

MEDLINE via PubMed	2016-06-09
1. "Trauma system" OR "Trauma network" OR "Trauma services" OR "Trauma health care" OR "Trauma model" OR "Trauma systems" OR "Trauma networks" OR "Trauma models" OR<br><br>"Injury network" OR "Injury system" OR "Injury services" OR "Injury health care" OR "Injury model" OR<br>"Injury systems" OR "Injury models" OR<br><br>"Trauma care system" OR "Trauma care network" OR "Trauma care organization" OR "Trauma care continuum" OR "Injury care system" OR "Injury care systems" OR<br><br>"Regional trauma care" OR "Regionalized trauma care" OR "Integrated trauma care"<br><br>2. animals[MeSH] NOT humans[MeSH]<br><br>3. #1 NOT #2	**5261 results**
**EMBASE**	**2016-08-09**
1. ("Trauma system" OR "Trauma network" OR “Trauma service” OR "Trauma services" OR "Trauma health care" OR "Trauma model" OR "Trauma systems" OR "Trauma networks" OR "Trauma models" OR "Injury network" OR “Injury networks” OR "Injury system" OR “Injury service” OR "Injury services" OR "Injury health care" OR "Injury model" OR "Injury systems" OR "Injury models" OR "Trauma care system" OR "Trauma care systems" OR "Trauma care network" OR "Trauma care organization" OR "Trauma care continuum" OR "Injury care system" OR "Injury care systems" OR "Regional trauma care" OR "Regionalized trauma care" OR "Regionalised trauma care" OR "Integrated trauma care")<br><br>2. ('animal'/exp NOT 'human'/exp)<br><br>3. #1 NOT #2	**9355 results**

exp: stands for explode and allows to search all the hierarchical terms related to a given concept within the Emtree thesaurus on EMBASE; MeSH: stands for Medical Subject Headings and allows to search any indexed article containing a given term in MEDLINE; NOT: boolean operator that specifies that the search must include everything except what follows the operator; OR: boolean operator that specifies to search any pair of adjacent terms or sets of terms
