# Supplementary material for: Impact of trauma system structure on injury outcomes: a systematic review protocol
Source: Syst Rev. 2017 Jan 21;6:12. doi: 10.1186/s13643-017-0408-8 (PMC5251247; doi:10.1186/s13643-017-0408-8)
Supplement: Additional file 3: — Data collection form. Form for extracting data from eligible articles. (PDF 145 kb) [file 13643_2017_408_MOESM3_ESM.pdf]

Data collection form*

**Title of review**	**INFLUENCE OF TRAUMA SYSTEM STRUCTURE ON INJURY OUTCOMES: A SYSTEMATIC REVIEW OF THE LITERATURE**
**Reviewer**	
**Date of review**	
**Form version**	1.0 (2016-04-08)
**Contact with author**	Date, reason, resolved
**Notes**	
**Study identification**	
**Report ID number**	last name first author - year of the reference
**Study ID number**	last name first author - year of the primary reference
**Title**	
**Author(s)**	
**Source1**	□ journal article □ abstract □ conference proceeding<br>other (e.g. unpublished data):
**Source 2**	□ Medline □ EMBASE □ CINHAL<br>□ BIOSIS □ Cochrane<br>□ Reference listing □ Hand search<br>Other (grey literature):
**Year**	
**Volume**	
**Page (start-end)**	
**Contact**	
**Country**	
**World Bank income classification**	□ low □ lower middle □ upper middle □ high
**Language**	
**Funding**	□ NA
**Notes**	

**Eligibility of study in the review**			
**Inclusion Criteria**	□ Listed injury outcome *(mortality, hospitalization, functional capacity, quality of life, burden of injury, adverse events, resource use, healthcare utilization)*		
	□ Evaluation of single or multiple organizational-level interventions for tertiary injury prevention		
**Notes**			
**Study details**			
**Setting**	*Country(ies)/province(s)/state(s); trauma system(s); number and level of centers*		
**Period of study**	*Dates start-end*		
**Study inclusion**	□ Age	Criteria:	□ NA
**criteria**	□ Injury severity	Criteria:	□ NA
	□ Injury type	Criteria:	□ NA
	□ Other	*List all other inclusion criteria*	□ NA
**Study exclusion criteria**	*List all exclusion criteria*		□ NA
**Ethics approval**	□ yes □ no □ unclear		
**Study design**	□ randomized controlled trial □ non randomized controlled trial □ Interrupted time series □ controlled before-after □ prospective cohort □ retrospective cohort □ uncontrolled quantitative study □ qualitative study<br>Other:		
**Notes**			
**Study data**			
**Primary data source**			□ NA
**Secondary data source**			□ NA
**Other data sources**			□ NA
**Notes**			
**Population characteristics**			
**Sample size (n)**	Patients :		□ NA
	Centers :		□ NA

**Age**	*e.g. n(%)≥65yoa, mean (SD), median (quartiles)*	□ NA
**Gender**	*n(%) male*	
**Mechanism**	*e.g. n(%) penetrating, MVC*	□ NA
**Injury severity**	*e.g. n(%) ISS>15, n(%) MAIS>3, ICISS*	□ NA
**Injury type**	*e.g. n(%) TBI; SCI; thoraco-abdominal; orthopedic; multiple injury types*	
**Notes**		
**Outcome (one per outcome)**		
**Outcome**		
**Definition**		
**Method of assessment**	*Diagnostic criteria or measurement tool used*	□ NA
**Timing of assessment**	*e.g. in-hospital*	□ NA
**Summary risk measure used**	□ prevalence □ incidence proportion □ incidence rate □ mean □ unclear □ none<br>Other :	
**Notes**		
**Intervention (one per intervention)**		
**Sample size**	Intervention group:	□ NA
	Control group:	□ NA
**Intervention**		
**Years since implementation**		
**Definition**		□ NA
**ACS recommended**	□ Yes □ No □ Unclear	□ NA
**WHO recommended**	□ Yes □ No □ Unclear	□ NA
**Type of comparison**	□ Across □ Within	
**Measure of association**	□ GMR □ OR □ RR □ HR<br>□ mean difference □ unclear □ none<br>Other:	
**Value of measure of association**		□ NA
**Standard error**		□ NA
**Confidence intervals**		□ NA
**Risk adjustment**	□ Yes □ No □ Unclear	
**Risk adjustment variables**		
**Notes**		

NA – not available
*Adapted from Cochrane Consumer and Communication Review Group Data extraction template
